# Thrombin, a Key Driver of Pathological Inflammation in the Brain

**DOI:** 10.3390/cells12091222

**Published:** 2023-04-23

**Authors:** Jaclyn Iannucci, Paula Grammas

**Affiliations:** 1 Department of Neuroscience and Experimental Therapeutics, School of Medicine, Texas A&M University, Bryan, TX 77807, USA; 2 The Roskamp Institute, Sarasota, FL 34243, USA

**Keywords:** neuroinflammation, neurodegeneration, Alzheimer’s disease, thrombin, therapeutic

## Abstract

Neurodegenerative diseases, including Alzheimer’s disease (AD), are major contributors to death and disability worldwide. A multitude of evidence suggests that neuroinflammation is critical in neurodegenerative disease processes. Exploring the key mediators of neuroinflammation in AD, a prototypical neurodegenerative disease, could help identify pathologic inflammatory mediators and mechanisms in other neurodegenerative diseases. Elevated levels of the multifunctional inflammatory protein thrombin are commonly found in conditions that increase AD risk, including diabetes, atherosclerosis, and traumatic brain injury. Thrombin, a main driver of the coagulation cascade, has been identified as important to pathological events in AD and other neurodegenerative diseases. Furthermore, recent evidence suggests that coagulation cascade-associated proteins act as drivers of inflammation in the AD brain, and studies in both human populations and animal models support the view that abnormalities in thrombin generation promote AD pathology. Thrombin drives neuroinflammation through its pro-inflammatory activation of microglia, astrocytes, and endothelial cells. Due to the wide-ranging pro-inflammatory effects of thrombin in the brain, inhibiting thrombin could be an effective strategy for interrupting the inflammatory cascade which contributes to neurodegenerative disease progression and, as such, may be a potential therapeutic target for AD and other neurodegenerative diseases.

## 1. Neuroinflammation: A Common Mechanism in Neurodegenerative Diseases

Neurodegenerative diseases are major contributors to death and disability worldwide. In 2016, neurological disorders were the leading cause of disability-adjusted life-years (DALYs; the sum of years of life lost) globally and the second leading cause of death [1,2]. These disorders, including Alzheimer’s disease (AD), Parkinson’s disease (PD), Multiple sclerosis (MS), and Amyotrophic lateral sclerosis (ALS), are pathological conditions defined by progressive and irreversible neuronal cell death and dysfunction [3]. While each of these diseases is characterized by unique patterns of neurodegeneration, as well as pathognomonic protein abnormalities which determine clinical presentation, neuroinflammation is an invariant feature of these disorders [4,5]. Exploring neuroinflammation in AD, a prototypical neurodegenerative disease, could help identify pathologic inflammatory mediators and mechanisms in other neurodegenerative diseases.

Pro-inflammatory cytokines and chemokines are significantly elevated in the brain of AD patients compared to healthy controls, as well as in animal models of AD [6,7,8,9]. These neuroinflammatory mediators appear early in the disease time course, suggesting their significant role in AD pathogenesis [8]. Animal studies show that chronic infusion of lipopolysaccharide (LPS) into the fourth ventricle of the rat brain reproduces the pathological changes found in the AD brain, supporting the role of neuroinflammation as a driver of AD pathology [10]. Additionally, a robust literature demonstrates the activation of inflammatory processes in brain areas closely associated with AD pathology, and activated microglia, thought to be key drivers of neuroinflammation, are found in or near the pathologic lesions of AD [11,12,13]. Circulating levels of systemic inflammatory mediators, such as interleukin (IL)-1β and tumor necrosis factor (TNFα), are chronically upregulated in AD, while an increased risk for AD is linked to systemic inflammatory conditions such as Type 2 diabetes, obesity, and rheumatoid arthritis [14,15]. Additionally, large-scale genome-wide association studies highlight brain-associated genetic variants linked to neuroinflammation in AD, including TREM2, CD33, and CR1 [16,17,18,19]. The idea that inflammatory mediators are linked to cognitive impairment is reinforced by experiments in apolipoprotein E (ApoE) knockout mice, which show induction of IL-6 and IL-8 in the brain microcirculation is associated with impaired cognition [20]. The link between neurovascular inflammation and cognitive function is further supported by data showing that reducing the expression of inflammation proteins in the cerebral microcirculation improves cognition in animal models of AD [21].

There is a multitude of experimental evidence suggesting that neuroinflammation acts as a critical pathological event, initiating and driving neurodegenerative processes associated with many neurological diseases. Therefore, identifying the key mediators of pathological inflammation in the brain is important to understanding and treating these devastating conditions. Elevated levels of the multifunctional inflammatory protein thrombin are commonly found in conditions that increase AD risk, including diabetes, atherosclerosis, and traumatic brain injury (TBI) [22,23,24,25]. Even though thrombin has been implicated in AD since the 1990s, when it was first detected in senile plaques and neurofibrillary tangles [26], the relevance of thrombin in AD pathology, and other neurodegenerative diseases, has not been widely explored.

## 2. Thrombin, a Key Driver of Neuroinflammation

Brain endothelial cells in AD express elevated levels of proteins that mediate cell adhesion and migration of inflammatory cells, including intracellular adhesion molecule (ICAM)-1, monocyte chemoattractant protein (MCP)-1, and CAP37 [27,28,29]. In addition, brain microvessels isolated from AD patient samples release higher levels of inflammatory mediators such as thrombin, nitric oxide (NO), TNFα, IL-1β, IL-6, IL-8, and matrix metalloproteinases (MMPs), compared to brain microvessels isolated from age-matched healthy controls [27,30,31]. The extensive number of chemokines and cytokines that are over-expressed in the vasculature in AD are consistent with an “activated proinflammatory endothelial phenotype” [32]. The multifunctional serine protease thrombin is a primary driver of endothelial activation and inflammation in both the periphery and the CNS in pathological conditions [33]. This pluripotent inflammatory mediator has a diverse array of cellular targets throughout the body and CNS, including endothelial cells, monocytes, neurons, astrocytes, pericytes, and microglia, through its activation of the protease-activated receptors (PARs) [34,35]. The importance of thrombin as a modulator of pathological inflammation in the brain is supported by data demonstrating that elevated levels of inflammatory cytokines evoked by systemic administration of LPS are decreased by inhibiting thrombin [36]. Furthermore, a direct role for thrombin in the inflammatory response characteristic of several neurodegenerative diseases has been suggested. PAR expression is increased in the substantia nigra of PD patients, and this increase is associated with the loss of dopaminergic cells, neuroinflammation, and oxidative stress [37,38,39]. In an animal model of MS, experimental autoimmune encephalomyelitis (EAE), elevated thrombin activity precedes the onset of neurological signs. Furthermore, thrombin activity was found to increase at the disease peak and was correlated with microglial activation, demyelination, axonal damage, and clinical severity [40]. In the same regard, expression of thrombin and PARs is increased in the AD brain [41,42].

The importance of thrombin as a regulator of cerebrovascular inflammation is further suggested by data in AD mice showing that thrombin inhibition causes a reduction in the production of inflammatory proteins. Furthermore, inflammation caused by hypoxia in brain endothelial cells is diminished by inhibiting thrombin [21]. These data showing the ability of thrombin to regulate brain vascular inflammation are important, as cerebral microcirculation in AD has been shown to produce thrombin. In this regard, thrombin message and protein levels are significantly increased in brain microvessels from AD patients and AD transgenic mice compared to controls [30,43]. In addition, immunoreactivity for the major thrombin inhibitor protease nexin 1 (PN1) is significantly decreased around blood vessels in AD, further suggesting that brain blood vessels are releasing thrombin in AD [44,45].

Finally, thrombin is widely appreciated as a central regulator of the coagulation cascade [46]. The coagulation cascade, consisting of both intrinsic and extrinsic pathways, involves sequential activation of clotting factors culminating ultimately in hemostasis. At the nexus of both intrinsic and extrinsic pathways is thrombin [47]. Thrombin cleaves fibrinogen, a soluble protein, into insoluble fibrin. Thrombin, through activation of Factor XIII, also affects the cross-linking of fibrin monomers to produce a firm fibrin clot and prevents clot fibrinolysis via activation of Factor XI [48]. Bidirectional communication between coagulation and inflammation is critical to the physiologic response to vascular injury [49]. Inflammation activates coagulation as a means of containing inflammatory damage, and coagulation-related factors, including thrombin, tissue factor (TF), and fibrin, can influence the extent and nature of inflammation [49,50]. For example, TF is pro-inflammatory because it can activate the PARs [51] and cause an increase in a variety of inflammatory proteins, including cytokines, chemokines, and adhesion proteins. Similarly, fibrin has been shown to regulate expression of inflammatory cytokines and chemokines and reactive oxygen species (ROS) in the brain [52,53,54]. Fibrin’s pro-inflammatory effects result in the activation of glia and disruption of blood–brain barrier (BBB) function [55]. Both fibrinogen and fibrin induce leukocyte migration and directly modulate the inflammatory response of leukocytes and endothelial cells [56].

Accumulating evidence demonstrates that coagulation cascade-associated proteins can initiate or drive inflammation in neurodegenerative diseases [33,38,52,57,58,59]. A role for the coagulation cascade in the development of MS pathology is supported by the finding that coagulation proteins are present in chronic active plaques in MS patients [60]. Similarly, in AD and AD animal models, fibrinogen co-localizes with amyloid plaques, pericyte loss, dystrophic neurites, and activated microglia [55,61]. The importance of this coagulation protein in the pathogenesis of AD is further suggested by data in AD mice, showing that fibrinogen depletion reduces cognitive dysfunction and AD-related pathology. It is important to note that thrombin is critical for fibrin formation, and therefore thrombin may function as an upstream mediator of the inflammatory effects of fibrin in the AD brain [46].

## 3. Thrombin-Mediated Inflammation: A Shared Mechanism among AD Risk Factors

Several diseases and conditions have been documented to increase the risk of developing AD. Among the most well-documented modifiable risk factors are atherosclerosis, diabetes, and TBI. A common thread among these risk factors is elevated levels of the pro-inflammatory protein thrombin.

### 3.1. Atherosclerosis

Given thrombin’s multiple effects on endothelial cells, which result in increased immune cell adhesion and inflammatory protein release, it is not surprising that thrombin is a driver of vascular pathology in atherosclerosis. Thrombin’s ability to promote atherosclerotic plaque formation reflects its key role as both a mediator of clot formation and inflammation [23,49,62]. Activation of PAR-1 by thrombin initiates signaling cascades in the vessel wall cell types, including endothelial cells, smooth muscle cells, and macrophages that are participatory in plaque genesis and development. In addition, the release of procoagulant factors, such as TF, which are evoked by thrombin, contributes to endothelial injury and plaque expansion [63].

Thrombin and its receptors (PARs) are elevated in atherosclerosis and are found around atherosclerotic plaques [64]. Thrombin is involved in the recruitment of circulating monocytes to atherosclerotic plaques by increasing expression of MCP-1, ICAM-1, vascular cell adhesion molecule (VCAM)-1, and platelet-derived growth factor (PDGF) [23,65,66,67], and increases in thrombin are associated with increased inflammation, angiogenesis, and cell proliferation in atherosclerotic models [23,68]. In addition, thrombin has pro-inflammatory effects on the vessel wall through its effects on NADPH oxidase and the NF-kB pathway. This activation of the NF-kB pathway leads to a number of downstream effects, including increased cell infiltration, increased expression of inflammatory molecules, promotion of hypercoagulability, and accelerated plaque development [69]. The observation of hypercoagulability in early atherosclerotic lesions supports the idea that the thrombin-stimulated release of procoagulant enzymes plays a fundamental role in the atherogenic process. Finally, genetic manipulations which increase thrombin exacerbate atherosclerosis-related pathology [70], while treatment with thrombin inhibitors reduces atherosclerosis-related pathology in animal models [71,72,73,74]. For example, thrombin inhibition attenuated atherosclerotic plaque formation and reduced both inflammation and PAR-1 expression in an ApoE-deficient mouse model of atherosclerosis [71].

### 3.2. Diabetes

Several lines of evidence support a role for thrombin in diabetes. Diabetic patients have increased thrombin levels in their blood, and high thrombin levels have been linked to poor diabetic control [75]. Hyperglycemia enhances thrombin generation leading to a hypercoagulable state in Type 2 diabetes [75,76,77]. Total thrombin generation and platelet reactivity are increased in Type 2 diabetes and older obese women [78]. An animal study examining the effects of obesity and metabolic syndrome, conditions frequently observed in diabetes, showed increased thrombin generation as early as 25 weeks of age [79]. Increased thrombin generation was also demonstrable in 7 to 10-week-old diabetic Zucker rats [80]. A study in the STZ rat demonstrated the importance of the thrombin pathway in a rat diabetic neuropathy model. Here, elevated thrombin activity was observed in the diabetic sciatic nerve correlating with the destruction of nodal histology and altered electrophysiological nerve conduction. Treatment with thrombin inhibitors ameliorated structural and electrophysiological deficits in diabetic animals [81].

Thrombin receptors are increased in the vasculature in the Type 1 diabetic animal model, streptozotocin (STZ) [82]. Thrombin, through its pro-inflammatory effects on vascular endothelial cells, is linked to endothelial dysfunction in diabetes [83]. In this regard, thrombin has been shown to increase oxidative stress in endothelial cells in diabetes via calcium-mediated intracellular pathways that regulate the transcription factor KLF14 and PLK1 kinase pathways [84]. In vitro, data support a direct role for thrombin in the noxious effects of glucose on endothelial cells. Treatment of brain endothelial cells with high glucose causes increased expression of inflammatory proteins (TNFα, IL-6, MMP-2, MMP-9) and ROS, an effect that is mitigated by inhibiting thrombin [85].

### 3.3. Traumatic Brain Injury (TBI)

Thrombin has been implicated in the pathological effects of TBI. Studies have identified elevated thrombin levels following TBI in both human and animal models [86,87,88,89,90], and it has been reported that neurons are exposed to elevated levels of thrombin following TBI [91,92]. A recent study found that thrombin levels rise in the first-hour post-trauma and again after 72 h [25]. Thrombin signaling activity after TBI has been associated with negative cognitive outcomes, including amnesia [93]. An increase in depressive behavior following TBI was also associated with thrombin-mediated down-regulation of hippocampal astrocyte glutamate transporters [94]. Thrombin’s negative effects after TBI have been linked to thrombin activation of both PAR-1 and PAR-4 [25,95].

Thrombin has been specifically implicated in inflammatory processes after TBI. Increases in thrombin are associated with the upregulation of adhesion molecules, including ICAM-1, by the cerebrovasculature, which is associated with increased peripheral immune cell migration into the brain [96,97,98]. Furthermore, elevated thrombin in TBI is associated with astrocyte activation and astrocyte-mediated inflammation [88], and inhibition of thrombin signaling after TBI via PAR-4 antagonism reduces expression of inflammatory genes related to the NF-κB signaling pathway [95].

## 4. Cellular Effects of Thrombin in the Brain: Implications for AD

Along with inflammation, thrombin has been associated with the classical pathological hallmarks of AD. Thrombin can trigger both tau and Aβ accumulation. Persistent thrombin signaling induces tau aggregation and hippocampal degeneration [99,100]. Thrombin induces secretion of AβPP, while Aβ promotes thrombin generation through Factor XII-mediated Factor XI activation [101]. Furthermore, thrombin delivered directly into the brain via intracerebroventricular injection results in significant neuropathology, enlargement of cerebral ventricles, increased TUNEL-positive cells, astrogliosis, ApoE fragments, and cognitive impairments [102,103]. Thrombin regulates the brain’s inflammatory response via pro-inflammatory activation of several cell types, including microglia, astrocytes, and especially endothelial cells of the BBB.

### 4.1. Microglia

Thrombin has been found to activate a pro-inflammatory state in microglia. The microglial response to thrombin includes several processes that modulate or contribute to microglia activation and/or apoptosis. Exposure of the microglial cell line BV2 to thrombin induces a pro-inflammatory response, including IL-1β release [104] and an increase in ROS and activation of the NLRP3 inflammasome [105], a component of the innate immune system. Primary microglia also take on a pro-inflammatory phenotype when stimulated by thrombin, characterized by increased production of cytokines, ROS, and NO [105,106,107,108]. Thrombin’s pro-inflammatory effects in microglia are directly linked to PAR-1 activation, which leads to the upregulation of microglial CD40 and increased TNFα production [109]. Additionally, thrombin activation of a TNFα/TNFR-dependent pathway downregulates expression of the mRNA species miR181c, which then promotes NF-κB target gene expression and related activity [110].

### 4.2. Astrocytes

Similar to microglia, astrocytes exhibit a shift towards a pro-inflammatory phenotype in response to thrombin. Thrombin induces MMP-9 expression and promotes cell migration via activation of the c-Src/Jak2/PDGFR/PI3K/Akt/PKCδ pathway in rat brain astrocytes [111]. Thrombin exposure can also alter astrocytic function, marked by disruptions in glutamate transport and altered stellation [88,112]. Furthermore, thrombin treatment in vivo increases GFAP expression in the rat hippocampus, indicating a likely increase in pro-inflammatory activation of astrocytes [103].

### 4.3. Endothelial Cells

**Figure 1:**
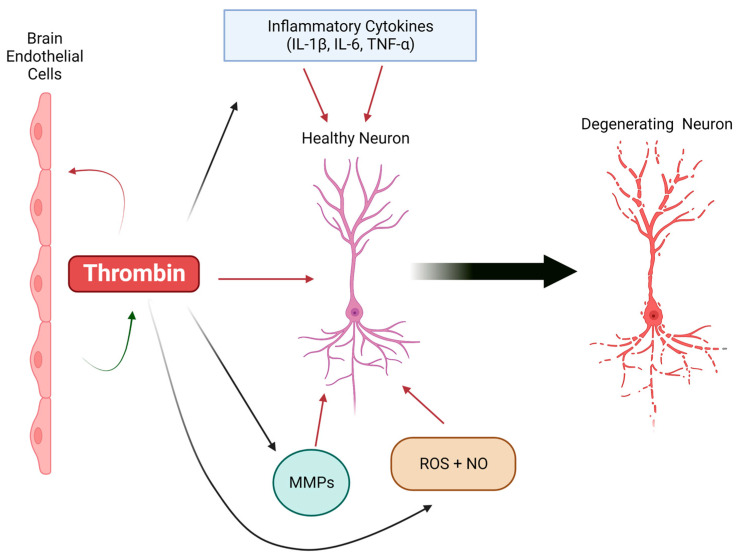
Thrombin-mediated neuroinflammation in neurodegenerative disease. Thrombin, produced by brain endothelial cells, acts as a regulator of neuroinflammatory processes in the brain, including inflammatory cytokines, matrix metalloproteinases (MMPs), reactive oxygen species (ROS), and nitric oxide (NO). Together, these inflammatory mediators can damage a healthy neuron and drive the neurodegeneration seen in Alzheimer’s disease (AD) and other neurodegenerative disorders.

Thrombin exerts many pro-inflammatory effects on endothelial cells. Thrombin modulates its effects on cerebrovascular endothelial cells via altered gene expression of pro-inflammatory mediators. Activation of brain endothelial cells by thrombin leads to increased expression and/or release of pro-inflammatory proteins, including ROS, NO, ICAM-1, VCAM-1, MCP-1, TNFα, IL-1, IL-6, and IL-8, as well as thrombin itself [97,98]. Additionally, thrombin stimulation modifies the organization of cell-to-cell junctions between endothelial cells and cytoskeletal actin filaments, leading to increased BBB permeability [98,113,114]. Finally, thrombin at the BBB is particularly problematic because brain endothelial cells can both release thrombin and respond to it via PAR-1 and PAR-3 [30,98]. Thus, thrombin can not only activate neighboring cells, but it can also act in an autocrine manner on the endothelium stimulating a noxious feed-forward cycle (Figure 1).

## 5. Thrombin: A Novel Inflammatory Therapeutic Target in AD

Since neuroinflammation contributes to the progression of AD and thrombin has wide-ranging pro-inflammatory effects in the brain, inhibiting thrombin could be an effective strategy for interrupting the inflammatory cascade which contributes to disease progression in AD.

Studies using anticoagulant therapies in both human patients and animal models support the idea that abnormalities in thrombin generation may promote AD-related pathology. In line with this notion, an open-label study of a hirudin (natural antithrombin anticoagulant) compound in eighty-four patients with mild-to-moderate AD found that hirudin plus donepezil reduced the rate of cognitive decline compared to donepezil alone [115]. Additionally, in a retrospective cohort study, the use of direct-acting anticoagulants, including dabigatran, was associated with a lower risk of dementia [116]. Although a placebo-controlled double-blind clinical trial of dabigatran in AD has yet to be performed, the possibility that anticoagulant therapy, specifically targeting thrombin, could be useful in AD is supported by a number of population-based studies that show reduced incidence of dementia in atrial fibrillation patients taking direct oral anticoagulants that target thrombin [116,117,118,119].

In transgenic AD mouse models, targeting thrombin has been shown to reduce brain inflammation as well as improve AD pathology. Treatment of transgenic AD mice with low molecular weight heparin enoxaparin reduces plaques and Aβ accumulation and improves spatial memory [120]. Treatment of 3xTgAD mice with the thrombin inhibitor dabigatran decreases cerebrovascular expression of inflammatory proteins, including thrombin, IL-6, MCP-1, and MMPs [21]. Long-term (1 year) dabigatran treatment of the TgCRND8 AD mouse model reduces neuroinflammation, amyloid plaques, and amyloid oligomers and preserves cognitive function [121]. Finally, treatment of a tau-based AD model (rTg4510) with a thrombin inhibitor decreases inflammation and oxidative stress-related and AD marker proteins (ratio of phospho-tau to total tau) [122]. Taken together, these data support the idea that inhibiting thrombin in the AD brain reduces inflammation and improves AD pathology and, as such, could be useful as part of a therapeutic regimen.

It should be noted that the use of any anticoagulant brings up safety concerns about the possibility of unwanted bleeding. Safety issues regarding the use of direct oral anticoagulants (DOACs), which include the direct thrombin inhibitor dabigatran, as well as Factor Xa inhibitors apixaban, edoxaban and rivaroxaban, have been extensively explored [116,123,124,125,126,127,128,129,130]. Treatment with DOACs was associated with lower rates of both stroke and systemic embolism when compared with warfarin [131]. In addition, both apixaban and dabigatran exhibited reduced rates of major bleeding, including gastrointestinal bleeding and intracranial hemorrhage, compared with warfarin [132]. A study in patients with an average age of 71.4 ± 8.6 years old showed that the use of dabigatran for over 30 months had minimal side effects [125].

A comparison of dabigatran and warfarin showed that dabigatran reduced the incidence of intracranial hemorrhage by 66% [132]. Dabigatran also exhibited an improved safety pattern than the Factor Xa-targeting rivaroxaban [133]. Rivaroxaban use was found to increase intracranial and extracranial hemorrhage compared to dabigatran [133]. The increased risk of bleeding with rivaroxaban compared to dabigatran may reflect the extent of blood–brain barrier penetration. Pharmacokinetic analysis of DOACs indicates that the greatest risk for adverse events is related to the level of BBB penetration. Evaluating the physicochemical and pharmacologic properties of DOACs reveals that BBB penetration is greatest with rivaroxaban, followed by apixaban, with dabigatran having the lowest potential risk for BBB penetration [134]. Finally, in mouse models of AD and cerebral amyloid angiopathy (CAA), treatment with dabigatran was not linked with increased intracerebral hemorrhage or microbleeds [135,136].

## 6. Conclusions

Drugs that provide symptomatic benefits for the treatment of AD, such as cholinesterase inhibitors and the glutamate antagonist memantine, have been available for decades [137]. However, developing effective therapeutic strategies for mitigating the progression of AD has proved challenging [138]. The pathogenesis of late-onset AD is complex and multifactorial; no single mechanism or pathologic mediator can account for AD progression. It is, therefore, not surprising that single-targeted amyloid-β-directed therapies have not shown significant clinical benefit to date. Recently, aducanumab, a monoclonal anti-Aβ oligomer antibody, received accelerated approval from FDA based on its ability to lower brain amyloid levels, although clinical benefit has yet to be determined [139], while late-stage lecanemab, a monoclonal antibody targeting Aβ aggregation, did appear to slow cognitive decline in people with early disease in a Phase 3 trial, but the effect was modest [140]. These data reinforce the idea that single-target therapies are not effective in mitigating the complex pathophysiology of AD.

Inflammation has long been appreciated as a central mechanism contributing to disease progression in AD. Therefore, targeting inflammation is a rational approach to AD prevention and therapy [141]. In this regard, long-term non-steroidal anti-inflammatory drug (NSAID) treatment is also associated with both reduced Aβ deposition in mouse models of AD and reduced number of plaque-associated microglia [142]. Retrospective epidemiological studies in humans suggest that a wide variety of NSAIDs may significantly reduce one’s lifetime risk of developing AD [143,144,145,146]. A recent meta-analysis of sixteen studies demonstrates that present or previous use of NSAIDs decreases the relative risk of AD [147]. However, despite these epidemiological data, anti-inflammatory placebo-controlled trials have yielded negative results.

A growing consensus in the AD treatment landscape argues for an innovative approach to AD therapy [148]. Following the example of other diseases such as cancer and HIV, parallel administration of two drugs that target different pathways could be employed, as well as using repurposed drugs as add-on treatments to existing standard-of-care protocols. Targeting the multifunctional inflammatory protein thrombin in conjunction with therapies directed at other AD targets (amyloid, tau) could be an innovative and novel therapeutic strategy in the fight against this devastating disease.

## Data Availability

No new data were created or analyzed in this study. Data sharing is not applicable to this article.
